# Determination of Lipoxygenase, CYP450, and Non-Enzymatic Metabolites of Arachidonic Acid in Essential Hypertension and Type 2 Diabetes

**DOI:** 10.3390/metabo12090859

**Published:** 2022-09-13

**Authors:** Guillaume Feugray, Tony Pereira, Michèle Iacob, Lucile Moreau-Grangé, Gaëtan Prévost, Valéry Brunel, Robinson Joannidès, Jérémy Bellien, Thomas Duflot

**Affiliations:** 1 CHU Rouen, Department of General Biochemistry, Normandie University, F-76000 Rouen, France; 2 UNIROUEN, INSERM U1096, Normandie University, F-76000 Rouen, France; 3 CHU Rouen, Department of Pharmacology, F-76000 Rouen, France; 4 CHU Rouen, Department of Endocrinology, Diabetes and Metabolic Diseases, Normandie University, F-76000 Rouen, France; 5 CHU Rouen, CIC-CRB U1404, F-76000 Rouen, France; 6 UNIROUEN, INSERM U1239, Normandie University, F-76000 Rouen, France

**Keywords:** type 2 diabetes, hypertension, oxylipins, arachidonic acid, epoxyeicosatrienoic acids, hydroxyeicosatrienoic acids, dihydroxyeicosatrienoic acids, hyperglycemic clamp, hyperinsulinemic clamp

## Abstract

Type 2 diabetes (T2D) and hypertension (HTN) are common risk factors of cardiovascular diseases (CVD) characterized by chronic low-grade systemic inflammation and impaired endothelial function. This study aimed to assess whether levels of non-enzymatic, lipoxygenase (LOX)- and cytochrome P450 (CYP)-derived arachidonic acid (ARA) metabolites, which are known regulators of vascular homeostasis, are affected by HTN and T2D. For this objective, 17 plasma level derivatives of ARA were quantitated by chromatography coupled with mass spectrometry in 44 patients (12 healthy, 8 HTN, 7 T2D, and 17 HTN + T2D). Effects of hyperglycemic and hyperinsulinemic clamps on ARA metabolite levels were assessed in seven healthy subjects. No significant differences in the plasma levels of ARA metabolites were observed for T2D patients compared with healthy volunteers. HTN was associated with an alteration of ARA metabolite correlation patterns with increased 20-, 19-, 15-, and 8-hydroxyeicosatrienoic acid (HETE). A decrease of 20-HETE was also observed during both hyperglycemic and hyperinsulinemic clamps. Additional experiments are needed to assess whether the modulation of HETE metabolites in HTN may be of interest. Furthermore, although not affected by T2D, it remains to investigate whether the decrease of 20-HETE observed during clamps may be related to the regulation of glucose tolerance and insulin signaling.

## 1. Introduction

Type 2 diabetes (T2D) and hypertension (HTN) are common risk factors of cardiovascular diseases (CVD) and the main causes of mortality in those populations especially when combined [1]. Both T2D and HTN are associated with chronic low-grade systemic inflammation characterized by an elevation in the systemic concentrations of pro-inflammatory mediators, such as cytokines and C-reactive protein (CRP) [2,3,4]. Of note, T2D- and HTN-induced CVD are also related to vascular tone dysfunction [5,6]. In the vascular system, physiological levels of reactive oxygen species (ROS), inflammatory mediators, and nitric oxide (NO) are essential for normal vascular functions, including endothelial homeostasis and smooth muscle cell contraction [7]. The balance between them is altered in T2D and HTN and strong evidence suggests that lipid mediators play a key role [8,9]. 

Amongst them, arachidonic acid (ARA), an omega-6 polyunsaturated fatty acid and its associated metabolites are considered as biological active compounds modulating vascular inflammation as well as vasomotor tone with either vasodilator [10,11] or vasoconstrictive [12] properties.

**Figure 1:**
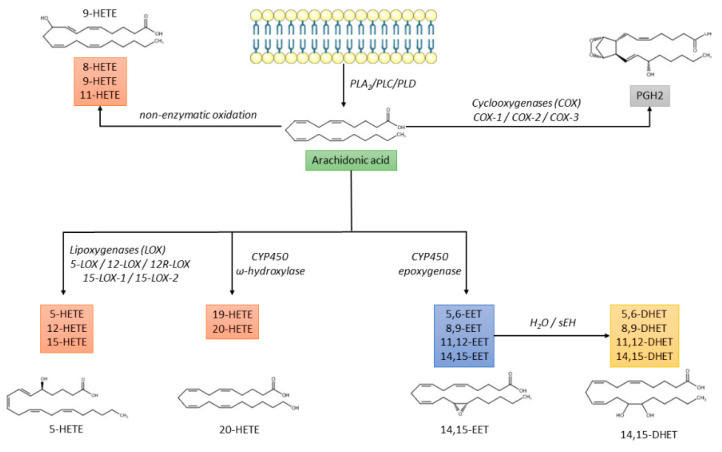
Biochemical pathways of arachidonic acid metabolism. CYP450: cytochrome P450; DHET: dihydroxyeicosatrienoic acid; EET: epoxyeicosatrienoic acid; HETE: hydroxyeicosatetraenoic acid; PGH2: prostaglandin H2; PL: phospholipase; sEH: soluble epoxide hydrolase.

ARA metabolism is complex, including three enzymatic pathways and a non-enzymatic oxidative pathway:

1. The cyclo-oxygenase (COX) pathway is responsible for the production of prostaglandin H2 (PGH2), which is further converted into prostaglandins (PGD2, PGE2, PGF2α, PGI2) or thromboxane A2 (TXA2) [13];
2. The lipoxygenase (LOX) pathway that produces hydroxyeicosatetraenoic acids (HETEs), leukotrienes, and lipoxins;
3. The cytochrome P450 (CYP) pathway that promotes the synthesis of HETEs and epoxyeicosatrienoic acids (EETs), which are further converted by soluble epoxide hydrolase (sEH) into dihydroxieicosatrienoic acids (DHETs) [14];
4. The non-enzymatic free radical oxidation pathway leading to the synthesis of 8-, 9-, and 11-HETE [15] (Figure 1).

Interestingly, ROS promotes ARA release through cytosolic phospholipase A2 activation as well as cyclo-oxygenase 2 (COX-2) expression leading to the production of PGE2 and PGD2. On the contrary, ROS can react with NO forming peroxynitrite that inactivates prostaglandin I synthase, suppressing the production of prostaglandin I2 (PGI2) [16]. Evidence suggests that eicosanoids are important mediators associated with blood pressure and T2D [17,18,19]. Since eicosanoids are usually low abundant lipids (nanomolar range) and more than 90% of the whole plasma oxylipins are esterified, these compounds are mainly quantitated after a saponification process to avoid sensitivity issues [20,21], but the clinico-biological relevance of free and/or esterified eicosanoids is still not fully elucidated. 

In this context, the aim of the present study was to quantitate free ARA-derived metabolites in order to investigate non-enzymatic, LOX, and CYP metabolic activity in healthy subjects and patients with HTN and/or T2D. In addition, hyperglycemic and hyperinsulinemic clamps were performed in healthy volunteers to assess the proper impact of the elevation in glucose and/or insulin levels on these lipid mediators. Biological approaches based on the quantitation of these factors under free form were performed.

## 2. Materials and Methods

### 2.1. Chemicals and Reagents

Methanol (MeOH), ethyl acetate (EA), and water of HPLC grade were purchased from Carlo Erba (Fontenay aux-Roses, France). Formic acid was purchased from VWR chemicals (Leuven, Belgium). 5-HETE, 9-HETE, 11-HETE, 15-HETE, 8,9-EET, 11,12-EET, 14,15-EET, 5,6-DHET, 8,9-DHET, 11,12-DHET, 14,15-DHET, 14,15-EETd_11_, 14,15-DHETd_11_, and 15-HETEd_8_ were purchased from Bertin technologies (Montigny-le-Bretonneux, France). Acetic acid was purchased from Carlo Erba (Fontenay-aux-Roses, France). Protein LoBind^®^ (1.5 mL) and standard Eppendorf (1.5 mL) tubes were purchased from Eppendorf (Hamburg, Germany). Standard Eppendorf microtubes (1.5 mL) were purchased from Eppendorf (Hamburg, Germany). Chromacol tubes were purchased from Thermo scientific (Langerwehe, Germany). Chromatographic Kinetex^®^ C18 column (50 mm L × 3 mm I.D., 2.6 μm) was purchased from Phenomenex (Le Pecq, France). Oasis HLB-SPE-columns (3 mL, 60 mg, 30 μm particles) were purchased from Waters (Guyancourt, France).

### 2.2. DHETs, HETEs, and EETs Quantitation

#### 2.2.1. Sample Preparation

A 4-F catheter with a 5 mL syringe was inserted into the forearm cephalic vein, when accessible, allowing local blood sampling in the venous return for the quantification of DHETs, EETs, and HETEs. Blood samples were immediately transferred on a prechilled lithium heparinate tube, centrifuged 5 min at 4500× *g* (+4 °C), snap frozen in liquid nitrogen, and stored at −80 °C until analysis.

Free oxylipins were extracted from individual plasma samples using a deproteinized step prior to a solid phase extraction (SPE). In the first step, 10 μL of IS solution in MeOH (30 ng/mL of 14,15-DHETd11, 14,15-EETd11, and 15-HETEd8) and 10 µL of MeOH were added to 500 µL of plasma. Then, 1 mL of MeOH was added and the sample was vortexed for deproteneization. After centrifugation (5 min, 20,000× *g*), the supernatant was loaded to a preconditioned Oasis HLB-SPE-column. The column was washed with 6 mL MeOH/water (5/95, *v*/*v*) and the cartridge was dried for 20 min. Oxylipins were eluted by gravity into glass tubes with 0.5 mL MeOH and 1.5 mL EA. Then, eluted compounds in MeOH/EA were evaporated under nitrogen for 20 min at 50 °C. The residue was reconstituted with 50 µL of MeOH, vortexed, transferred into an autosampler vial and injected (10 µL) into the UHPLC–MS-MS system.

#### 2.2.2. LC-MS/MS Conditions

Oxylipin assays were performed on a LC–MS/MS system consisting of the following Shimadzu^®^ modules (Shimadzu Corporation, Marne-la-Vallée, France): a binary pump consisting of coupling two isocratic pumps Nexera LC30AD, an automated sampler SIL-30AC, a column oven CTO-20AC and a triple-quadrupole mass spectrometer LCMS-8060 operating in the negative ion mode. 

Chromatographic separation was achieved on a Kinetex^®^ C18 column (50 mm L × 3 mm I.D., 2.6 μm) maintained at 50 °C and a gradient of (A) water at 0.01% acetic acid and (B) methanol at a flow rate of 0.600 mL/min as follows: 0.0–0.5 min, 10% (B); 0.5–2.0 min, 10 to 70% (B); 2.0–5.0 min, 70 to 75% (B); 5.0–5.1 min, 75 to 98% (B); 5.1–6.9 min, 98% (B); 6.9–7.0 min, 98% to 10% (B); 7.0–8.0 min, 10% (B).

The source interface parameters and common settings were as follows: interface voltage: −3 kV; nebulizing gas flow: 3 L/min; heating gas flow: 10 L/min; drying gas flow: 10 L/min; interface temperature: 400 °C; DL (desolvation line) temperature: 250 °C; heat block temperature: 500 °C; collision gas pressure 300 kPa.

Detection and quantification were performed by scheduled-MRM (Multiple Reaction Monitoring) using a pause time of 3 ms and individual dwell times to achieve sufficient points per peak. Isobaric compounds of 14,15-DHET; 14,15-EET, and 15-HETE were used as internal standards (IS) for each family of compounds (Supplementary Table S1).

#### 2.2.3. Method Validation

In order to provide reliable quantitation of the selected compounds, the following have been performed:

1. Calibration curves were obtained by spiking the standards at increasing concentrations (0, 10, 20, 50, 100, 200, 500, 1000, 2000, and 5000 pg/mL) with a fixed concentration of the IS (30 ng/mL for both 14,15-DHET-d_11_, 14,15-EET-d_11_ and 15-HETE-d_8_) using a different matrix: phosphate-buffered saline (PBS), BSA (PBS + bovine serum albumin 8%), and plasma;
2. Calibration curve linearity, lower limit of quantification (LLOQ), and carryover were assessed according to FDA guidelines on validation of bioanalytical methods for each analyte [22]. Parallelism between the three matrices were also investigated;
3. Sample recovery (RE), matrix effect (ME), and process efficiency (PE) were determined according to Matuszewski et al. [23]. Calibration curves (0, 10, 20, 50, 100, 200, 500, 1000 pg/mL) were prepared in MeOH as a reference matrix (Set 1). Bovine serum albumin (BSA), phosphate-buffered saline (PBS) and plasma matrix were spiked post-extraction (Set 2) and pre-extraction (Set 3). BSA and PBS were spiked with the same calibrator levels as Set 1. Plasma matrix was spiked with three calibrator levels (200, 500, and 1000 pg/mL) and baseline signal due to the presence of endogenous analytes was subtracted to obtain the true spiked signal. This allowed for calculation of RE (Set 3/Set 2 × 100), ME (Set 2/Set 1 × 100), and PE (Set 3/Set 1 × 100);
4. Oxylipins adsorption during the deproteinization step was performed using standard eppendorf (1.5 mL), Lobind^®^ eppendorf (2 mL), and Chromacol (4 mL). PBS was used as a surrogate matrix and spiked with 10 ng/mL of each compound. Analyses were performed in triplicate;
5. Comparative analysis of lithium heparinate and ethylenediaminetetraacetic acid (EDTA) on compound concentrations was performed. Samples were drawn at the same time for the same subject for a 1-on-1 comparison and the concentration of each oxylipin was assessed. Analyses were performed from five subjects randomly selected from the study and for whom an aliquot of plasma from EDTA and heparin tubes remained.

### 2.3. Population

This study was performed in a total of 44 subjects. Subjects who smoked more than five cigarettes per day, with cardiac and/or cerebrovascular ischemic vascular disease, heart failure, or impaired renal function (estimated glomerular filtration rate < 60 mL/min/1.73 m^2^) were excluded from the study. Patients with T2D must not have a HbA1c > 9.4 mmol/L (7.5%), obtained with lifestyle management and standard hypoglycemic agents except insulin. Patients with essential hypertension with and without T2D were included. 

### 2.4. Hyperglycemic and Hyperinsulinemic Clamps

Hyperglycemic and hyperinsulinemic euglycemic clamps were performed in seven healthy subjects and explored on two separate occasions after a 12 h overnight fasting. Briefly, a venous catheter was inserted in a large vein at the antecubital fossa of the dominant arm, allowing a variable infusion rate of a 20% glucose solution alone or combined with continuous regular insulin administration (Actrapid^®^ 100 UI/mL, Novo Nordisk: 250, 200, 150, 100 mU/m^2^ of body surface area per min for 2 min each and then 80 mU/m^2^ of body surface area per min) to achieve and maintain either a target steady-state capillary glucose concentration of 11 mmol/L (200 mg/dL) for the hyperglycemic clamp or 5.5 mmol/L (100 mg/dL) for the hyperinsulinemic clamp, without change in glucose infusion rate for at least 30 min. Blood sampling was performed before infusion and at glycemic steady-state to determine the variation of free DHETs, EETs, and HETEs.

### 2.5. Statistical Analysis

Statistical analyses and figures were performed using R v4.1.0 [24] and the following packages: *RVAideMemoire* v0.9-81-2 [25], *ggsci* v2.9 [26], *ggpubr* v0.4.0 [27], *ggplot2* v3.3.5 [28], *rstatix* v0.7.0 [29], *emmeans* v1.6.1 [30], *nlme* v3.1.152 [31], *corrplot* v0.88 [32], *reshape2* v1.4.4 [33], and *igraph* v1.2.6 [34]. Continuous and count data were expressed as median [interquartile range or IQR] and n (%), respectively. For baseline characteristics of the study population, *p*-values were computed using Fisher’s exact test for nominal data and ANOVA for continuous data followed by post hoc pairwise comparison tests in case of significance with corrections for multiple testing (false discovery rate, Benjamini & Hochberg). Pairwise Fisher’s exact test and Tukey’s honest significant difference method were used for nominal and continuous data, respectively. 

For the surrogate matrix analyses, linear mixed models were performed with an interaction term between concentration and matrix to compare slope coefficients.

Oxylipin concentrations were investigated by multiple linear regression using HTN and T2D as predictors to discriminate the impact of each compound on physio(patho)logical status compared to healthy controls. 

Oxylipin patterns were assessed using Pearson’s correlation matrix. Correlation networks were built for significant relationships (*p* < 0.05) with a correlation coefficient threshold of 0.5 (r ≥ 0.5).

Associations between statistically significant plasma levels of oxylipins compared to healthy subjects and risk factors of CVD (systolic blood pressure, age, body mass index, sex, and glycemia) were assessed by simple linear regression.

DHETs, EETs, and HETEs variation during hyperglycemic and hyperinsulinemic euglycemic clamps was assessed using paired *t*-tests.

Raw data for process efficiency, calibration curves, clinical study, and clamps as well as R code are available as Supplementary Files S1–S5, respectively.

## 3. Results

### 3.1. DHETs, HETEs and EETs Analytical Method

The developed LC-MS/MS method allowed for quantification of 14 compounds (4 dihydroxy-, 7 hydroxy-, and 3 epoxy-ARA derivatives) within a run of 8 min. Of note, specific transitions were needed due to coelution phenomena for the following pairs of analytes: 8- and 12-HETE, 5-HETE and 14,15-EET, and 8,9- and 11,12-EET. Furthermore, similar fragmentation patterns were observed between HETEs and EETs requiring chromatographic separation of these analytes (Supplementary Figure S1). 

Method validation exhibited an LLOQ of 10 pg/mL for DHETs, 14,15- and 11,12-EET; 20 pg/mL for 20-, 15-, and 11-HETE; 50 pg/mL for 8,9-EET, 19-, 8-, and 5-HETE, and 100 pg/mL for 12-HETE. ULOQ were fixed at 5000 pg/mL for 8,9-EET; 1000 pg/mL for DHETs, 11,12-EET, 20-, 19-, 15-, 12-, and 11-HETE and 500 pg/mL for 14,15-EET, and 8- and 5-HETE based on expected concentrations. Blank samples injected after three ULOQ samples revealed no carryover effect. Calibration curves for all analytes respected FDA calibration curve acceptance criteria (calibrators within ±20% and ±15% of the theoretical concentrations for LLOQ and other calibrators, respectively).

Coefficient of variation of the slopes for each calibration curve performed in triplicate were acceptable (CV < 25%). Parallelism investigations revealed significant differences of slope coefficients when compared with plasma matrix for 5,6-DHET, 11,12-EET, 8,9-EET, 20-HETE,19-HETE and 5-HETE for PBS matrix and for 11,12-DHET, 8,9-DHET, 5,6-DHET, 14,15-EET, 11,12-EET, 8,9-EET, and 20-HETE for BSA matrix (Supplementary Figure S2 and Table S2).

The method yielded RE ranging from 35.9 to 79.3% for BSA, from 35.3 to 95.6% for PBS, and from 32.3 to 76.7% for plasma matrix. ME between the BSA (ranging from 53.9 to 109.2%) and PBS (ranging from 80.5 to 102.9%) matrix were similar with an overall lower coefficient of variation for PBS. Interestingly, the plasma matrix exhibited signal suppression with ME ranging from 12.7 to 91.3%. As a whole, PE was lower in plasma (ranging from 6.0 to 57.6%), compared with the BSA (ranging from 33.4 to 81.1%) and PBS (ranging from 34.6 to 99.2%) matrix. The use of an internal standard prevented differences between the biological and surrogate matrix for 14,15-EET and 20-, 19-, 15-, 12-, 11-, 8-HETE, while true concentrations could not be achieved for the other analytes (Table 1).

Analysis of oxylipin recovery showed an average 2.05 (min: 1.22 max: 2.62) and 2.14 (min: 0.88 max: 3.02) higher recovery than standard Eppendorf microtubes for Lobind^®^ Eppendorf microtubes and Chromacol tubes, respectively (Supplementary Table S3).

Comparison of lithium heparinate and EDTA tubes for blood deposition after syringe sampling revealed higher concentrations of 11,12-EET, 14,15-EET, 5-HETE, and 12-HETE when blood was transferred in lithium heparinate with a heparine-to-EDTA ratio higher than 1.5 (Supplementary Table S4).

### 3.2. Baseline Popoulation Characteristics

Amongst the 44 subjects included in the study, 12 healthy volunteers (control), 8 HTN patients, 7 T2D patients, and 17 HTN + T2D patients were enrolled corresponding to 12 control subjects, 25 patients with HTN, and 24 patients with T2D. No differences were observed between groups for age, sex ratio, smoking status, systolic blood pressure (SBP), diastolic blood pressure (DBP), mean blood pressure (MBP) heart rate, HDL cholesterol, triglycerides (TG), Hb1Ac, creatinemia, or antihypertensive and hypoglycemic agents. 

Fasting glycemia was higher in the T2D group compared with the control group and the HTN group. 

Body mass index (BMI) and fasting glycemia were higher in the hypertensive diabetic (HTN + T2D) group compared with the control group and the HTN group. Finally, HTN + T2D subjects were more frequently treated with statins and had a lesser LDL cholesterol compared with the control group (Table 2).

### 3.3. Analysis of Oxylipin Profiles

**Figure 2:**
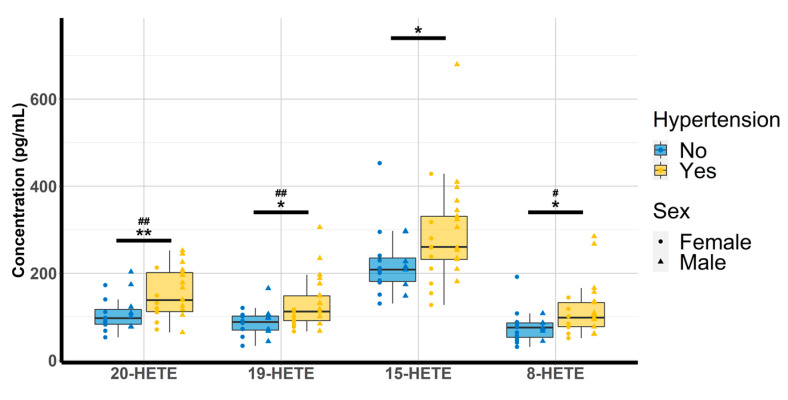
Evaluation of HTN and gender effect on 20-HETE, 19-HETE, 15-HETE, and 8-HETE (n = 44). HETE: hydroxyeicosatetraenoic acid. * *p* < 0.05, ** *p* < 0.01 (hypertension); ^#^ *p* < 0.05 ^##^ *p* < 0.01 (sex).

Oxylipin concentrations were quantitated and analyzed according to the physio(patho)logical status of each subject. Amongst the 14 compounds, 8,9-EET could not be investigated due to concentrations below the lower limit of quantitation (LLOQ) and 12-HETE was discarded due to concentrations above the upper limit of quantitation (ULOQ). The multiple linear regression revealed that none of the free oxylipins analyzed were significantly altered by T2D. However, patients with HTN exhibited higher concentrations of 20-HETE (98.9 [81.7–148.1] vs. 138 [112–202]; *p*-value = 0.009), 19-HETE (89.4 [71.8–99.5] vs. 112 [91–148]; *p*-value = 0.012) 15-HETE (213 [202–254] vs. 261 [232–331]; *p*-value = 0.042) and 8-HETE (75.7 [66.8–86.6] vs. 98.2 [77.3–132.7], *p*-value = 0.026) (Table 3 and Figure 2). 

Analyses between oxylipins and risk factors of CVD revealed that higher concentrations of 19-, 15-, and 8-HETE were associated with higher SBP (*p* = 0.039, *p* = 0.023, and *p* = 0.030, respectively). Furthermore, higher concentrations of 20-, 19-, and 8-HETE were observed in men compared with women (*p* = 0.001, *p* = 0.001, and *p* = 0.034, respectively; Figure 2).

Correlation network analyses revealed two distinct nodes in control subjects. The first gathered 14,15-EET, 11,12-EET, and 5-HETE and the second included all other compounds except 5,6-DHET.

**Figure 3:**
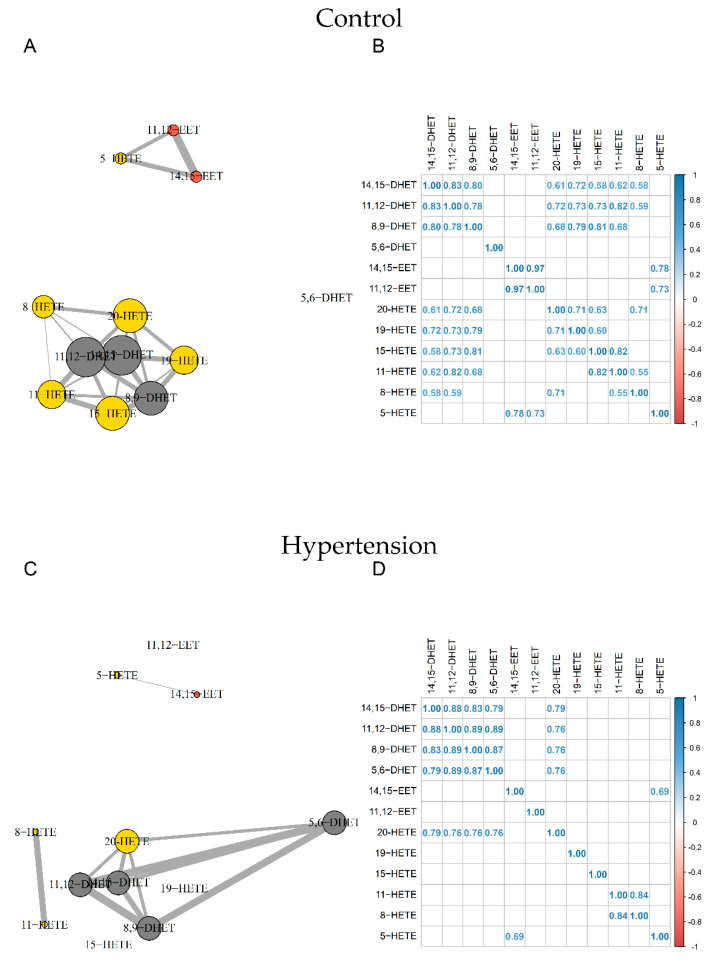
Correlation network and correlation matrix for Control group (**A**,**B**) and HTN (**C**,**D**). EETs, HETEs, and DHETs are shown in orange, yellow, and grey, respectively. Sizes of nodes and edges are proportional to the number of correlations and the strength of the correlation, respectively (n = 44). DHET: dihydroxyeicosatrienoic acid; EET: epoxyeicosatrienoic acid; HETE: hydroxyeicosatetraenoic acid; HTN: hypertension.

For HTN subjects, the strong relationship between 11,12-EET and 14,15-EET (R^2^ = 0.97) and between 11,12-EET and 5-HETE (R^2^ = 0.73) were disrupted. We also observed a loss of relationships between 19-, 15-, 11-, 8-HETE, and DHETs. Furthermore, whereas 5,6-DHET had no significant relationship with other compounds in the control group, it was correlated with other DHETs and 20-HETE in the HTN group (Figure 3).

### 3.4. Clamp Investigations

**Figure 4:**
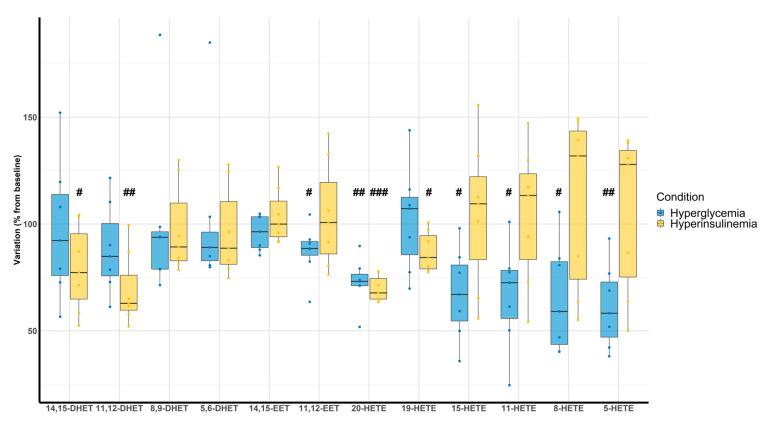
Evaluation of DHETs, EETs, and HETEs variation according to hyperglycemic (blue) and hyperinsulinemic euglycemic (yellow) clamps compared with baseline (n = 7). ^#^ *p* < 0.05, ^##^ *p* < 0.01, *^###^ p* < 0.001.

Hyperglycemic clamps were associated with a decrease of 11,12-EET (87 ± 12%, *p* = 0.035), 20-HETE (73 ± 11%, *p* < 0.001), 15-HETE (67 ± 21%, *p* = 0.007), 11-HETE (67 ± 24%, *p* = 0.011), 8-HETE (65 ± 25%, *p* = 0.011), and 5-HETE (61 ± 20%, *p* = 0.002) compared with the baseline. On the other hand, hyperinsulinemic clamps were associated with a decrease of 14,15-DHET (79 ± 20%, *p* = 0.036), 11,12-DHET (76 ± 17%, *p* = 0.003), 20-HETE (70 ± 6%, *p* < 0.001), and 19-HETE (87 ± 10%, *p* = 0.012) (Figure 4).

## 4. Discussion

### 4.1. Analytical Method

The present analytical method aimed to accurately quantify 14 ARA derivatives, including monohydroxy-, dihydroxy-, and epoxy- metabolites produced from either enzymatic or non-enzymatic pathways. The outputs from our analytical method validation depicted satisfying results with a run of 8 min and a high sensitivity ranging from 10 to 50 pg/mL for all analytes except 12-HETE. It also raised several challenges that need to be addressed in order to consider absolute quantitation. First of all, these compounds are prone to adsorption phenomena using standard Eppendorf microtubes mainly due to hydrophobic interactions between the standard polymer surface and the compounds [35]. The use of Lobind^®^ Eppendorf microtubes or glass tubes, such as chromacol may prevent this phenomenon resulting in an approximately 2-fold increase in the signal obtained by mass spectrometry. Secondly, blood collection is a critical step and our analysis revealed huge differences between EDTA and lithium heparin tubes with an increase in HETEs and a decrease in DHETs when blood was deposited in lithium heparin tubes. Interestingly, it has previously been demonstrated that heparin may induce phospholipase 2 activity [36] as well as platelet activation, resulting in an increase in LOX expression leading to higher HETEs levels [37]. Of note, in our study, samples were drawn in lithium heparin tubes and that could explain why 12-HETE exhibited concentrations above the ULOQ. Last but not least, absolute quantitation of endogenous compounds is not trivial. Since an analyte-free biological matrix is difficult to obtain and there is a lack of stable-isotope-labeled analog for each compound, the choice has been made to evaluate two different surrogate matrices that could mimic the plasma matrix: PBS and BSA. Of note, absolute quantitation requires that the response function of the surrogate matrix truly depicts what happens in the biological matrix, which was assessed by process efficiency [23] and parallelism [38]. Our results raised the need of harmonized procedures between laboratories as previously demonstrated [39] since our approach, based on classical oxylipin analysis workflow (deproteinization, SPE and evaporation), revealed either differences in PE and/or a lack of parallelism for some compounds (Supplementary Figure S2) despite the use of stable-isotope-labeled analog. Finally, the analytical method developed did not explore specific enantiomer abundance (R- and S-HETEs and DHETs, cis- and trans-EETs); it could have been worth determining if the significant changes in oxylipin plasma levels were due to an increase in enzymatic activity or auto-oxidation processes since both can occur for the same regioisomer.

### 4.2. Oxylipin Analysis in Pathological Status

In this study, subjects were carefully selected and patients (HTN and/or T2D) benefited from the best usual care following the latest recommendations in the management of risk factors (BMI, LDL-cholesterol, blood pressure, and HbA1c) using therapeutic drugs (statins, hypoglycemic, and antihypertensive agents). This is especially important to highlight since despite those treatments, long-term outcome of these patients could be of poor prognosis, indicating that new therapeutic targets are needed. Of note, 17 patients exhibited both HTN and T2D leading to a lack of power in the statistical analysis using the 4 groups depicted in Table 1. The choice was made to compare oxylipin profiles using a multiple linear regression model using both HTN and T2D as predictors in order to maintain sufficient power while assessing the impact of each compared to healthy subjects. As a consequence, T2D alone did not seem to impact the oxylipin profiles in this population but this may also be explained by the fact that T2D patients were well controlled on their glycemic status and therefore may be less at risk for profound alterations of ARA metabolism. Regarding HTN patients, a significant increase in several HETE isoforms (8-, 15-, 19-, and 20-HETE) has been pointed out. Interestingly, elevated 20-HETE was previously associated with arterial stiffness and systolic hypertension in a murine model of hypertension with metabolic syndrome via matrix metalloproteinase 12 (MMP12) activation [40]. 20-HETE is a known potent vasoconstrictor of several arteries with EC_50_ in the nM range. The underlying mechanism involved PKC, MAPK, src-type tyrosine kinase, and rho kinase pathways that all contribute to the regulation of the vascular tone [41]. Interestingly, biological and physiological properties of 19-HETE were not deeply investigated but it seems to exhibit a protective effect against cardiac hypertrophy [42]. The most plausible hypothesis is that 19-HETE may be considered as an antagonist of the 20-HETE receptor [43]. Since both 19- and 20-HETE are synthetized by CYP ω-hydroxylases, the increase in 19-HETE may be linked to a compensatory mechanism opposing the deleterious effects of 20-HETE. The observed increase in 15-HETE also appeared relevant because it has been demonstrated that 15-LOX, responsible for the synthesis of 15-HETE from ARA, is histologically localized to the vascular endothelium [44]. Interestingly, 15-HETE displays vasodilatory properties at low concentrations [45] but contractions at higher concentrations [46]. This could be attributed to the fact that HETEs could bind with low or high affinity to different prostanoid receptors, such as prostaglandin and/or thromboxane receptors leading to opposite effects [47]. Of note, HTN is a risk factor for the development of atherosclerosis and it has been demonstrated that atherosclerotic arteries produce increased amounts of 15-HETE [48]. Finally, we also observed an increase in 8-HETE in HTN patients compared with healthy volunteers. Unlike the mouse for which 15-LOX-2 allows its synthesis, this compound is only produced by radical oxidation in humans. It has been demonstrated that 8-HETE promotes hypertrophy in human ventricular cardiomyocytes through MAPK NF-κB dependent mechanism [49] and is increased in patients with acute coronary syndrome [50]. Since it is well known that the prevalence of HTN is higher in men than in woman and that men are more likely to develop CVD at an earlier age than women, the fact that we observed higher concentrations of 20-HETE, 19-HETE, and 8-HETE in males suggests that receptors of these compounds may be interesting targets for therapeutic purposes. Recently, large-scale sequencing of human genome has identified a rare variant of G-protein coupled receptor GRP75, a specific target of 20-HETE, associated with resistance to weight gain and improved glycemic control in a high fat model [51]. This receptor stimulates G_αq/11_ dissociation and increased inositol phosphate leading to angiotensin-converting enzyme overexpression and endothelial dysfunction. Interestingly, a mouse model lacking GRP75 prevented blood pressure elevation, ACE expression, endothelial dysfunction, and smooth muscle contractility induced by 20-HETE. Thus, GRP75 provides a new therapeutic target to limit adverse effects induced by 20-HETE for metabolic syndrome [52]. Taken together, looking at each HETE individually does not seem quite informative since they can bind to several receptors exhibiting opposite effect as well as be produced by a compensatory mechanism. Thus, correlation network analysis allowed for investigating all compounds as a whole, looking at the relationship between them for control, HTN, and T2D subjects. The fact that the number of significant correlations decreased in HTN patients when compared with the control strengthened the hypothesis that oxylipin homeostasis is deeply affected during a physiopathological condition. In this study, the loss of relationships between HETEs and DHETs as well as HETEs themselves supposed a dysregulation of enzymes involved in oxylipins synthesis or degradation and/or an increase in reactive oxygen species-driven HETEs synthesis.

### 4.3. Clamps

Since T2D patients included in this study were treated by hypoglycemic agents, hyperglycemic and hyperinsulinemic euglycemic clamps were performed on 7 out of the 12 controls in order to specifically address the effect of glucose metabolism abnormalities. Interestingly, hyperglycemia led to a significant decrease of all HETEs isoforms except 19-HETE. On the other hand, hyperinsulinemia coupled with euglycemia only induced changes in 14,15-DHET, 11,12-DHET, and 20-HETE. The most important changes concern 20-HETE, which decreased by 27% and 30% for hyperglycemia and hyperinsulinemia, respectively. Looking at the literature, 20-HETE not only affects the vascular tone but also plays an important role in insulin signaling [53]. Indeed, 20-HETE is a highly efficacious agonist of FFAR1, a long-chain fatty acid receptor that induces glucose-stimulated insulin secretion in pancreatic β-cells [54]. On the other hand, cytochrome P450 4F2 (CYP4F2) transgenic mice with high levels of 20-HETE production exhibited attenuated glucose-stimulated insulin secretion [55]. Here, the decrease in 20-HETE in both hyperglycemic and hyperinsulinemic euglycemic clamps strongly supports the hypothesis that 20-HETE is an important regulator of insulin signaling and glucose tolerance. Importantly, clamps were performed on healthy volunteers without insulin resistance suggesting a potential inhibitory effect on cytochrome P450 ω-hydroxylase driven by the increase in insulin release from hyperglycemic and hyperinsulinemic clamps through a negative feedback loop, which is abolished in case of uncontrolled insulin resistance [56].

## 5. Conclusions

This study suggests that an altered ARA metabolism was observed in patients with HTN. The increase in 8-,15-, 19- and/or 20-HETE may contribute to vascular tone dysfunction. Additional experiments are warranted using modulators of ARA metabolism and downstream signaling, such as specific inhibitors of LOX or CYP450 ω-hydroxylase as well as prostanoid receptor antagonists, to decipher if these approaches may restore cardiovascular homeostasis in HTN with the expected results of improving the prognosis of patients. Although not affected by T2D, clamps exploration confirmed the tight relationship between 20-HETE, insulin signaling, and glucose metabolism, which needs further investigation due to the risk of false positive results caused by the small sample size.

## Figures and Tables

**Table 1:** Recovery, matrix effect and process efficiency values for DHETs, EETs, and HETEs from BSA, PBS, and plasma.

Analyte and IS	Recovery			Matrix Effect			Process Efficiency			Analyte/IS Process Efficiency		
	BSA	PBS	Plasma	BSA	PBS	Plasma	BSA	PBS	Plasma	BSA	PBS	Plasma
14,15-DHET	35.9 ± 10.2 (28%)	35.3 ± 6.0 (17%)	32.3 ± 9.4 (29%)	109.2 ± 30.6 (28%)	98.5 ± 18.1 (18%)	54.3 ± 6.9 (13%)	37.1 ± 7.9 (21%)	34.6 ± 7.2 (21%)	17.1 ± 3.6 (21%)	82.6 ± 13.0 (16%)	88.6 ± 18.2 (21%)	122.0 ± 29.1 (24%)
11,12-DHET	47.7 ± 10.5 (22%)	45.7 ± 6.8 (15%)	43.7 ± 11.1 (25%)	90.7 ± 10.7 (12%)	88.8 ± 7.6 (9%)	61.9 ± 9.3 (15%)	43.1 ± 10.2 (24%)	40.5 ± 6.3 (16%)	26.5 ± 4.8 (18%)	96.0 ± 16.9 (18%)	103.7 ± 15.6 (15%)	189.1 ± 40.8 (22%)
8,9-DHET	65.2 ± 12.4 (19%)	74.5 ± 17.4 (23%)	53.5 ± 5.1 (10%)	87.6 ± 11.5 (13%)	89.6 ± 9.8 (11%)	60.0 ± 8.3 (14%)	57.0 ± 120 (21%)	65.8 ± 11.2 (17%)	32.2 ± 6.2 (19%)	128.5 ± 27.0 (21%)	169.1 ± 30.8 (18%)	233.5 ± 66.3 (28%)
5,6-DHET	68.0 ± 9.8 (14%)	79.8 ± 12.5 (16%)	67.9 ± 16.0 (24%)	99.1 ± 13.0 (13%)	92.4 ± 8.9 (10%)	71.3 ± 6.9 (10%)	66.5 ± 7.1 (11%)	73.6 ± 12.4 (17%)	47.8 ± 9.2 (19%)	150.1 ± 21.7 (14%)	189.3 ± 35.2 (19%)	336.7 ± 52.4 (16%)
14,15-EET	68.3 ± 10.8 (16%)	83.0 ± 11.9 (14%)	67.1 ± 13.1 (19%)	91.5 ± 10.5 (12%)	93.2 ± 10.1 (11%)	72.0 ± 7.0 (10%)	61.9 ± 9.0 (15%)	77.1 ± 12.4 (16%)	48.3 ± 10.0 (21%)	82.5 ± 14.0 (17%)	85.7 ± 12.4 (15%)	92.9 ± 15.5 (17%)
11,12-EET	66.5 ± 30.4 (46%)	95.6 ± 44.9 (47%)	42.1 ± 12.3 (29%)	53.9 ± 20.3 (38%)	80.5 ± 22.0 (27%)	21.6 ± 5.6 (26%)	33.4 ± 12.7 (38%)	72.0 ± 26.8 (37%)	9.3 ± 4.6 (49%)	44.4 ± 16.4 (37%)	80.2 ± 30.8 (38%)	18.1 ± 9.0 (50%)
8,9-EET	59.4 ± 23.4 (39%)	71.8 ± 14.6 (20%)	43.6 ± 16.6 (38%)	60.6 ± 14.3 (24%)	88.5 ± 18.0 (20%)	12.7 ± 4.8 (38%)	37.3 ± 16.8 (45%)	63.5 ± 16.3 (26%)	6.0 ± 4.3 (71%)	48.5 ± 18.6 (38%)	72.8 ± 19.1 (26%)	11.5 ± 7.7 (67%)
20-HETE	79.3 ± 27.9 (35%)	79.3 ± 23.6 (30%)	61.2 ± 13.7 (22%)	89.8 ± 13.2 (15%)	90.3 ± 19.4 (22%)	72.4 ± 13.4 (19%)	70.5 ± 23.9 (34%)	68.8 ± 14.5 (21%)	43.5 ± 9.3 (21%)	91.1 ± 30.6 (34%)	77.9 ± 11.1 (14%)	74.8 ± 20.0 (27%)
19-HETE	62.2 ± 22.1 (36%)	72.3 ± 13.9 (19%)	60.2 ± 18.7 (31%)	106.1 ± 30.8 (29%)	91.8 ± 10.4 (11%)	82.7 ± 15.3 (18%)	65.6 ± 30.5 (46%)	65.7 ± 11.1 (17%)	47.6 ± 8.6 (18%)	82.3 ± 33.4 (41%)	77.3 ± 17.0 (22%)	80.6 ± 12.3 (15%)
15-HETE	67.8 ± 14.0 (21%)	77.8 ± 15.3 (20%)	64.9 ± 15.9 (24%)	99.7 ± 19.0 (19%)	92.5 ± 10.9 (12%)	90.5 ± 10.3 (11%)	68.1 ± 25.0 (37%)	71.1 ± 11.4 (16%)	57.6 ± 9.8 (17%)	89.8 ± 40.0 (45%)	80.9 ± 9.2 (11%)	98.4 ± 20.6 (21%)
12-HETE	73.0 ± 8.7 (12%)	80.1 ± 7.1 (9%)	57.9 ± 17.3 (30%)	94.1 ± 14.6 (15%)	97.2 ± 7.7 (8%)	91.3 ± 26.0 (28%)	68.0 ± 8.7 (13%)	77.7 ± 7.3 (9%)	50.8 ± 16.9 (33%)	88.1 ± 14.7 (17%)	89.6 ± 10.8 (12%)	87.4 ± 29.0 (33%)
11-HETE	77.1 ± 10.6 (14%)	85.8 ± 10.4 (12%)	64.5 ± 12.6 (20%)	105.4 ± 15.2 (14%)	102.9 ± 12.9 (13%)	77.2 ± 15.7 (20%)	81.1 ± 15.1 (19%)	88.2 ± 14.1 (16%)	49.3 ± 11.5 (23%)	104.8 ± 15.6 (15%)	100.6 ± 14.8 (15%)	85.4 ± 24.9 (29%)
8-HETE	72.8 ± 12.1 (17%)	77 ± 9.5 (12%)	62.1 ± 20 (32%)	89.2 ± 16.8 (19%)	86.9 ± 12 (14%)	72.4 ± 20.1 (28%)	64.1 ± 12.4 (19%)	66.8 ± 12.4 (19%)	43 ± 12 (28%)	82.4 ± 15.9 (19%)	77.9 ± 12.5 (16%)	75.3 ± 27.1 (36%)
5-HETE	75.6 ± 11 (15%)	78.6 ± 5.6 (7%)	76.7 ± 19.3 (25%)	100.6 ± 12.2 (12%)	100.3 ± 8.2 (8%)	53.4 ± 11 (21%)	75.5 ± 11 (15%)	78.7 ± 7.1 (9%)	39.6 ± 7.1 (18%)	97.5 ± 16.4 (17%)	92.7 ± 14.3 (15%)	67.3 ± 11.4 (17%)
14,15-DHET-d_11_	43.6 ± 7.5 (17%)	39.6 ± 4.7 (12%)	24.6 ± 7.2 (29%)	102.3 ± 9.1 (9%)	100.6 ± 6.9 (7%)	62.5 ± 19 (30%)	45 ± 7.4 (16%)	39.3 ± 5.3 (14%)	14.2 ± 2.1 (15%)	-	-	-
14,15-EET-d_11_	76.8 ± 10.8 (14%)	89 ± 15.1 (17%)	72.2 ± 12.4 (17%)	98.8 ± 9.2 (9%)	103.5 ± 8.5 (8%)	73.2 ± 10.9 (15%)	76.1 ± 11.2 (15%)	90.9 ± 14.3 (16%)	52.4 ± 9.7 (18%)	-	-	-
15-HETE-d_8_	80.2 ± 14.1 (18%)	90.1 ± 11.4 (13%)	69.7 ± 8.2 (12%)	97.9 ± 10.4 (11%)	98.1 ± 6.8 (7%)	85.5 ± 9.6 (11%)	77.9 ± 12.4 (16%)	88.4 ± 12.8 (14%)	59.2 ± 6.9 (12%)	-	-	-

Data are expressed as mean ± s.d (CV%) performed in triplicate. BSA: bovine serum albumin matrix; CV: coefficient of variation; s.d.: standard deviation; IS: internal standards; PBS: phosphate buffered saline matrix.

**Table 2:** Demographics and clinico-biological characteristics of the study population.

Parameters	Control (n = 12)	HTN (n = 8)	T2D (n = 7)	HTN + T2D (n = 17)
Age, years	56 [53–61]	57 [54–61]	58 [55–63]	60 [58–66]
Male, n (%)	5 (42%)	4 (50%)	2 (29%)	11 (65%)
Body mass index (kg/m^2^)	25 [23–26]	25 [25–27]	26 [25–30]	31 [29–34] *^,†^
Smoking status, n				
Current/Past/Never	1/4/7	0/0/8	1/1/5	0/9/8
SBP, mmHg	125 [119–133]	140 [130–146]	129 [127–140]	135 [132–141]
DBP, mmHg	77 [74–80]	86 [82–90]	81 [79–89]	81 [72–91]
MBP, mmHg	92 [91–95]	106 [97–109]	95 [95–105]	100 [93–108]
Heart rate, bpm	64 [60–71]	58 [56–72]	71 [61–81]	72 [64–76]
LDL cholesterol, g/L	1.48 [1.37–1.66]	1.17 [1.07–1.24]	1.25 [1.02–1.38]	0.80 [0.62–1.04] *
HDL cholesterol, g/L	0.59 [0.48–0.70]	0.54 [0.47–0.69]	0.70 [0.47–0.73]	0.53 [0.40–0.61]
Triglycerides, g/L	0.74 [0.70–0.91]	0.88 [0.72–1.5]	0.99 [0.81–1.42]	1.00 [0.81–1.35]
Fasting glycemia, mg/dL	1.00 [0.88–1.00]	0.93 [0.90–1.01]	1.40 [1.30–1.58] *^,†^	1.35 [1.18–1.45] *^,†^
Insulinemia, pmol/L	55 [37–78]	71 [51–98]	69 [62–78]	90 [67–147] *
Hb1Ac, mmol/L	-	-	6.9 [6.7–7.4]	6.6 [6.2–6.9]
Creatinemia, µmol/L	70 [61–73]	75 [62–86]	67 [61–73]	65 [56–79]
Statins, n (%)	1 (8%)	2 (25%)	2 (29%)	13 (76%) *
Antihypertensive agents, n (%)				
ACEi/ARB	-	7 (88%)	-	12 (72%)
CCB	-	2 (25%)	-	11 (65%)
Beta-blokers	-	0 (0%)	-	2 (12%)
Diuretics	-	0 (0%)	-	7 (41%)
Hypoglycemic agents, n (%)				
Metformin	-	-	7 (100%)	14 (82%)
Sulfamides/glinides	-	-	1 (14%)	7 (41%)
DPP-4 inhibitors/GLP-1 agonists	-	-	2 (29%)	8 (47%)

Data are expressed as median [interquartile] or n (%). ACEi: angiotensin-converting enzyme inhibitors; ACR: albumin-to-creatinine ratio; ARB: angiotensin type 1 receptor blockers; CCB: calcium channel blockers; DBP: diastolic blood pressure; DPP-4: dipeptidyl peptidase-4; GLP-1: glucagon-like peptide-1; HTN: hypertension; MBP: mean blood pressure; SBP: systolic blood pressure; T2D: type 2 diabetes. * *p* < 0.05 vs. Control ^†^ *p* < 0.05 vs. HTN.

**Table 3:** Oxylipin concentrations according to physio(patho)logical status.

Analyte (pg/mL)	Control	HTN	T2D
14,15-DHET	134 [129–169]	156 [129–190]	152 [128–172]
11,12-DHET	134 [122–163]	149 [116–171]	120 [116–158]
8,9-DHET	66 [58–72]	68.3 [52.9–87.5]	71.1 [57.0–89.2]
5,6-DHET	114 [88–155]	108 [77–174]	110 [80–214]
14,15-EET	16.2 [11.8–19.2]	20.3 [15.4–40.2]	19.3 [14.7–42.2]
11,12-EET	19.7 [18.3–21.7]	25.0 [21.9–33.7]	23.2 [18.1–32.4]
8,9-EET	<LLOQ	<LLOQ	<LLOQ
20-HETE	98.9 [81.7–148.1]	138 [112–202] **	118 [95.9–184]
19-HETE	89.4 [71.8–99.5]	112 [91–148] *	108 [85.6–136]
15-HETE	213 [202–254]	261 [232–331] *	242 [196–325]
12-HETE	>ULOQ	>ULOQ	>ULOQ
11-HETE	95.9 [92.4–102.3]	107.0 [93.4–147.9]	103 [88.0–143]
8-HETE	75.7 [66.8–86.6]	98.2 [77.3–132.7] *	84.1 [61.5–106]
5-HETE	121 [112–189]	211 [142–270]	204 [138–239]

Data are expressed as median [interquartile] or n (%). DHET: dihydroxyeicosatrienoic acid; EET: epoxyeicosatrienoic acid; HETE: hydroxyeicosatetraenoic acid; HTN: hypertension; LLOQ: lower limit of quantitation; NA: not available; T2D: type 2 diabetes; ULOQ: upper limit of quantitation. Multiple linear regression was performed using HTN and T2D as predictors. n = 44, * *p*  <  0.05 vs. Control ** *p*  <  0.01 vs. Control.

## Data Availability

Data presented in this study as well as R code for statistical analyses, tables, and figures are available in Supplementary Materials.

## Supplementary Materials

The following supporting information can be downloaded at: https://www.mdpi.com/article/10.3390/metabo12090859/s1, Figure S1: Extracted ion chromatogram of PBS matrix spiked with 100 pg/mL of each compound with their specific mass transition and retention time; Figure S2: Calibration curves of analyzed oxylipins on plasma, PBS, and BSA matrix; Table S1: LC-MS/MS parameters for DHETs, EETs, HETEs and internal standards; Table S2: Comparison of the slope of the calibration curves according to the matrix; Table S3: Recovery rate of PBS spiked with 10 ng/mL according to deproteinization tube; Table S4: Comparison between lithium heparinate and EDTA on oxylipin concentrations; File S1: Process Efficiency File S2: Calibration curves data; File S3: Clinical study data; File S4: Clamps data; File S5: R code.
