# Supplementary material for: Determination of Lipoxygenase, CYP450, and Non-Enzymatic Metabolites of Arachidonic Acid in Essential Hypertension and Type 2 Diabetes
Source: Metabolites. 2022 Sep 13;12(9):859. doi: 10.3390/metabo12090859 (PMC9501142; doi:10.3390/metabo12090859)
Supplement: Supplementary file 1 [file metabolites-12-00859-s001.zip › Supplementary Tables.pdf]

Supplementary Table S1. LC-MS/MS parameters for DHETs, EETs, HETEs and internal standards.

Analyte	Mass transition		MS parameters				Adduct	Retention time (min)
	m/z (MS1)	m/z (MS3)	Dwell Time (msec)	Q1 Pre Bias (V)	CE	Q3 Pre Bias (V)		
14,15-DHET	337.20	207.20	17	12	18	15	$[M-H]^-$	4.167
11,12-DHET	337.20	167.10	17	12	18	12	$[M-H]^-$	4.395
8,9-DHET	337.20	127.20	17	12	20	14	$[M-H]^-$	4.489
5,6-DHET	337.20	145.05	17	12	16	11	$[M-H]^-$	4.840
14,15-EET	319.00	219.25	17	17	12	16	$[M-H]^-$	5.704
11,12-EET	319.20	208.10	10	11	12	11	$[M-H]^-$	5.941
8,9-EET	319.20	155.00	10	23	12	11	$[M-H]^-$	5.990
20-HETE	319.00	245.15	38	14	17	20	$[M-H]^-$	4.755
19-HETE	319.00	231.35	38	16	17	13	$[M-H]^-$	4.697
15-HETE	319.20	219.15	38	11	13	11	$[M-H]^-$	5.236
12-HETE	319.20	179.15	38	30	14	30	$[M-H]^-$	5.379
11-HETE	319.20	167.15	38	15	16	12	$[M-H]^-$	5.263
8-HETE	319.20	155.10	38	11	15	11	$[M-H]^-$	5.401
5-HETE	319.20	114.90	17	26	14	30	$[M-H]^-$	5.744
14,15-DHET-d_{11}	348.10	207.10	10	17	18	15	$[M-H]^-$	4.213
14,15-EET-d_{11}	330.10	219.25	10	16	12	16	$[M-H]^-$	5.642
15-HETE-d_8	327.10	226.25	10	16	14	16	$[M-H]^-$	5.178

Supplementary Table S2. Comparison of the slopes of the calibration curves according to the matrix.

Analyte	Plasma	PBS	BSA
14,15-DHET	0,00216 ± 0,00031	0,00238 ± 0,00028	0,00209 ± 0,00037
11,12-DHET	0,00253 ± 0,00062	0,00253 ± 0,00029	0,00194 ± 0,00026*†
8,9-DHET	0,00081 ± 0,00013	0,00077 ± 0,00009	0,00050 ± 0,00005*†
5,6-DHET	0,00170 ± 0,00034	0,00093 ± 0,00005*	0,00062 ± 0,00008*‡
14,15-EET	0,0186 ± 0,0018	0,0167 ± 0,0027	0,0130 ± 0,0008*‡
11,12-EET	0,00044 ± 0,00017	0,00110 ± 0,00015*	0,00088 ± 0,00009*‡
8,9-EET	0,00035 ± 0,00025	0,00133 ± 0,00026*	0,0007 ± 0,00017*
20-HETE	0,00043 ± 0,00003	0,00061 ± 0,00008*	0,00054 ± 0,00008*
19-HETE	0,00051 ± 0,00008	0,00065 ± 0,00013*	0,00059 ± 0,00011
15-HETE	0,00220 ± 0,00024	0,00266 ± 0,00035	0,00242 ± 0,00050
12-HETE	0,00246 ± 0,00077	0,00307 ± 0,00048	0,00314 ± 0,00062
11-HETE	0,00858 ± 0,00092	0,00996 ± 0,00164	0,00987 ± 0,00200
8-HETE	0,00171 ± 0,00012	0,00208 ± 0,00012	0,00174 ± 0,00002
5-HETE	0,00155 ± 0,00035	0,00227 ± 0,00036*	0,00183 ± 0,00006

Data are expressed as mean ± standard deviation. * $P < 0.05$ vs. plasma †$P < 0.05$ vs. PBS, ‡$P < 0.05$ vs. BSA. BSA: bovine serum albumin, PBS: phosphate-buffered saline.

Supplementary Table S3. Recovery rate of PBS spiked with 10 ng/mL according to deproteinization tube

Analyte	Standard eppendorf (Area)	Chromacol (Area)	Lobind® eppendorf (Area)
14,15-DHET	10500092 ± 2434693 (23%)	17816564 ± 3535152 (20%)	24902899 ± 4714984 (19%)
11,12-DHET	5778393 ± 557955 (10%)	9422037 ± 1638894 (17%)	11407911 ± 3074228 (27%)
8,9-DHET	1403792 ± 260491 (19%)	2668709 ± 414299 (16%)	3328670 ± 541536 (16%)
5,6-DHET	1554017 ± 184718 (12%)	2823235 ± 487060 (17%)	2802168 ± 383358 (14%)
14,15-EET	5076407 ± 448163 (9%)	11754670 ± 1926462 (16%)	8352584 ± 1197625 (14%)
11,12-EET	385158 ± 65824 (17%)	899617 ± 328555 (37%)	677568 ± 205449 (30%)
8,9-EET	277662 ± 78668 (28%)	678557 ± 406847 (60%)	799683 ± 228336 (29%)
20-HETE	404586 ± 45141 (11%)	825689 ± 140290 (17%)	832081 ± 140737 (17%)
19-HETE	526012 ± 27857 (5%)	1002883 ± 183593 (18%)	831065 ± 102427 (11%)
15-HETE	978295 ± 78624 (8%)	2694306 ± 369284 (14%)	2858493 ± 69104 (2%)
12-HETE	1215713 ± 69371 (6%)	2649338 ± 638721 (24%)	3339454 ± 434012 (13%)
11-HETE	3007745 ± 361534 (12%)	7796443 ± 1608635 (21%)	9073193 ± 1114071 (12%)
8-HETE	1478025 ± 78921 (5%)	3457326 ± 721683 (21%)	3947060 ± 240355 (6%)
5-HETE	1016582 ± 86904 (9%)	2537499 ± 509498 (20%)	2778363 ± 304264 (11%)
14,15-DHETd11	9077321 ± 916723 (10%)	11146754 ± 2299663 (21%)	12314215 ± 4377150 (36%)
14,15-EETd11	1217819 ± 99085 (8%)	1825700 ± 325808 (18%)	1082065 ± 21066 (2%)
15-HETEd8	1612311 ± 339478 (21%)	2886719 ± 312984 (11%)	2488547 ± 271853 (11%)

Data are expressed as mean ± s.d. (CV%) performed in triplicates. CV: coefficient of variation; s.d.: standard deviation.

Supplementary Table S4. Comparison between lithium heparinate and EDTA sampling on oxylipins concentrations

Analyte	Heparine (pg/mL)	EDTA (pg/mL)	Heparine-to-EDTA ratio (CV%)
14,15-DHET	196 ± 71	200 ± 67	0.98 ± 0.07 (7.2%)
11,12-DHET	204 ± 88	198 ± 76	1.05 ± 0.23 (22.3%)
8,9-DHET	79 ± 23	81 ± 24	1.00 ± 0.26 (26%)
5,6-DHET	131 ± 39	126 ± 42	1.06 ± 0.19 (18%)
14,15-EET	27 ± 11	20 ± 16	1.98 ± 1.32 (66%)
11,12-EET	30 ± 7	26 ± 15	1.51 ± 0.99 (65%)
8,9-EET	48 ± 4	44 ± 6	1.09 ± 0.15 (13%)
20-HETE	143 ± 56	132 ± 69	1.13 ± 0.26 (23%)
19-HETE	105 ± 35	119 ± 36	0.88 ± 0.06 (7%)
15-HETE	314 ± 83	320 ± 97	0.99 ± 0.11 (11%)
12-HETE	1672 ± 1222	689 ± 363	2.73 ± 2.59 (95%)
11-HETE	74 ± 17	77 ± 21	0.98 ± 0.17 (17%)
8-HETE	75 ± 14	71 ± 16	1.06 ± 0.08 (7%)
5-HETE	168 ± 13	132 ± 49	1.50 ± 0.81 (54%)

Data are expressed as mean ± s.d. (CV%) performed in 5 replicates. CV: coefficient of variation; s.d.: standard deviation.
