# Supplementary figures and images for: Determination of Lipoxygenase, CYP450, and Non-Enzymatic Metabolites of Arachidonic Acid in Essential Hypertension and Type 2 Diabetes
Source: Metabolites. 2022 Sep 13;12(9):859. doi: 10.3390/metabo12090859 (PMC9501142; doi:10.3390/metabo12090859)

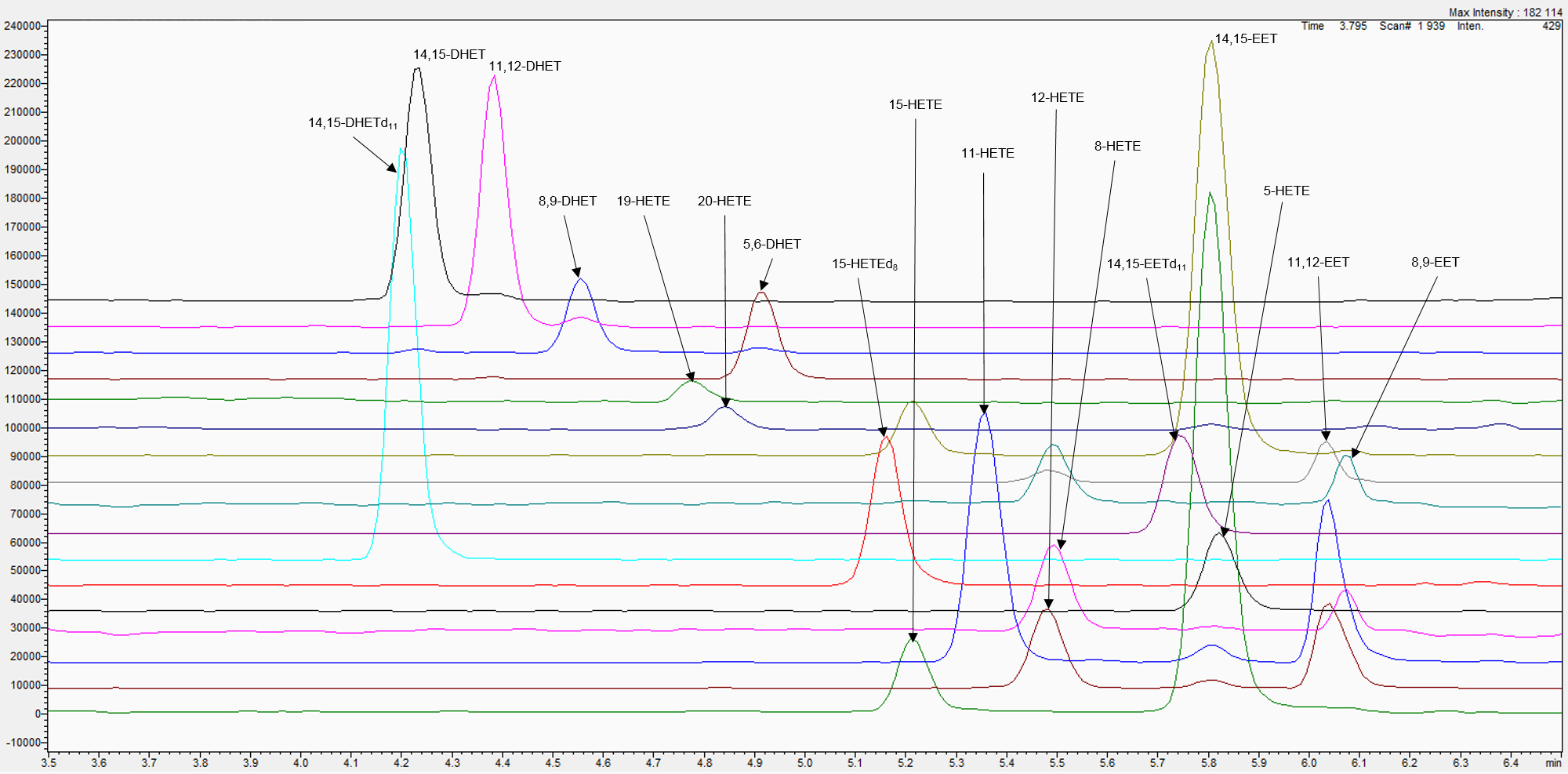

Supplement: Supplementary file 1 [file metabolites-12-00859-s001.zip › Supplementary Figure S1.png]
